# Active prokaryotic and eukaryotic viral ecology across spatial scale in a deep-sea brine pool

**DOI:** 10.1093/ismeco/ycae084

**Published:** 2024-06-14

**Authors:** Benjamin Minch, Morgan Chakraborty, Sam Purkis, Mattie Rodrigue, Mohammad Moniruzzaman

**Affiliations:** Department of Marine Biology and Ecology, Rosenstiel School of Marine, Atmospheric, and Earth Science, University of Miami, Miami, FL 33149, United States; Department of Marine Geosciences, Rosenstiel School of Marine, Atmospheric, and Earth Science, University of Miami, Miami, FL 33149, United States; Department of Marine Geosciences, Rosenstiel School of Marine, Atmospheric, and Earth Science, University of Miami, Miami, FL 33149, United States; OceanX, New York, NY 10018, United States; Department of Marine Biology and Ecology, Rosenstiel School of Marine, Atmospheric, and Earth Science, University of Miami, Miami, FL 33149, United States

**Keywords:** brine pool viruses, deep-sea viruses, marine virus ecology, virus–host interactions, marine viral diversity, NEOM brine pools

## Abstract

Deep-sea brine pools represent rare, extreme environments, providing unique insight into the limits of life on Earth, and by analogy, the plausibility of life beyond it. A distinguishing feature of many brine pools is presence of thick microbial mats that develop at the brine–seawater interface. While these bacterial and archaeal communities have received moderate attention, viruses and their host interactions in these environments remain underexplored. To bridge this knowledge gap, we leveraged metagenomic and metatranscriptomic data from three distinct zones within the NEOM brine pool system (Gulf of Aqaba) to reveal the active viral ecology around the pools. We report a remarkable diversity and activity of viruses infecting microbial hosts in this environment, including giant viruses, RNA viruses, jumbo phages, and Polinton-like viruses. Many of these form distinct clades—suggesting presence of untapped viral diversity in this ecosystem. Brine pool viral communities exhibit zone-specific differences in infection strategy—with lysogeny dominating the bacterial mat further away from the pool’s center. We linked viruses to metabolically important prokaryotes—including association between a jumbo phage and a key manganese-oxidizing and arsenic-metabolizing bacterium. These foundational results illuminate the role of viruses in modulating brine pool microbial communities and biogeochemistry through revealing novel viral diversity, host associations, and spatial heterogeneity in viral dynamics.

## Introduction

Deep-sea brine pools represent one of Earth’s most extreme environments due to their hypersaline anoxic conditions, and low pH [1]. Their formation is largely caused by the stable accumulation of hypersaline brine generated from the interaction between seawater and buried salt deposits that accumulate in seabed depressions [2, 3]. So far, these unique environments are known to exist in only three major water bodies: the Gulf of Mexico, the Mediterranean, and the Red Sea [4–6]. Within their respective bodies of water, brine pools are rare and small in comparison to their host basin, ranging in size from hundreds of square meters to a few square kilometers [7]. Most Brine pools are found in deep-sea depressions, but a few have been found in shallower coastal-shelf waters of the Red Sea [8].

Though small, brine pools represent oases of life for both microorganisms and macrofauna. These environments stand in contrast to the rest of the deep benthos where life is scarcer than the photic zone [9, 10]. Brine pools teem with life, hosting dense microbial mats, as well as various species of bivalves, shrimp, and fish [7, 11]. Hence, brine pools have become key model ecosystems for studying the limits of life on our planet as well as the potential for life beyond it [12–14]. Specifically of interest is the comparability of these deep-sea brine pools to the conditions of the subsurface oceans on the icy moons that orbit Jupiter and Saturn [12, 15, 16].

Early work advocated that these deep-sea brine pools were sterile, but this notion was drastically reversed after groundbreaking phylogenetic studies of the bacteria that thrive in association with the brine [17]. The majority of microbial life in the brine pools exists at the interface between the anoxic brine and the surrounding seawater, as dense bacterial mats are often observed surrounding the pools [10, 18–20]. A hypothesized factor contributing to the abundant growth is the density gradient created at the brine-seawater interface that can act as a trap for organic and inorganic materials from seawater [21, 22]. Such trapping makes the brine a rich source of nutrients for specific microbes to harness for growth. Along with having a nutrient sink, the microbes that inhabit the sediment in and around a brine pool also exhibit vertical and spatial stratification due to different physiochemical gradients of chemicals such as manganese, sulfate, and potassium, promoting diverse microbial metabolic strategies [23, 24]. Another key aspect of the stratification of the microbial communities is the abrupt shift from oxic to anoxic conditions in the pool, as there is little to no mixing within the pool [25]. This interface creates niches for a plethora of methanogenic and sulfate-reducing bacteria to flourish [26].

Bacterial communities in diverse environments are usually associated with the presence of viral populations, and it seems this holds true for even the most extreme of environments. Studies of brine pool sediments in the Mediterranean, for instance, demonstrate high levels of viral infection, proposing viral lysis as a key top-down control of prokaryotes in the brine pool sediments [27]. This is not to say that eukaryotic grazing is not present, as zooplankton and fish have been observed feeding on the thick particles at the brine-seawater interface [7, 28] and many species of fungi, dinoflagellates, and ciliates have been discovered around the pools [29].

Although viruses are typically thought of predominantly as cell-lysis agents, not all viruses adopt the same infection or replication strategy. Viral reproduction occurs primarily through either lytic or lysogenic infection, with the quantitative importance of each of these processes varying greatly throughout the ocean [30]. Within the lytic cycle, viruses infect hosts, replicate inside of their cells and eventually lyse the cell to release more viruses into the environment. The lysogenic cycle involves a temporal separation between infection and lysis as the virus will integrate into the host genome. Viruses can switch between the lysogenic cycle and lytic cycles, which usually occurs from a variety of factors including changes in nutrients [31] or environmental stressors such as UV radiation [32]. Bacterial populations associated with lysogenic viruses have been hypothesized to have a competitive advantage as lysogenic viruses may protect against infection from homologous viruses [33] and confer beneficial traits in the form of auxiliary metabolic genes (AMGs) [34]. Although lysogeny was initially thought of as a survival strategy for viral communities with low host abundance [35], the recent “Piggyback-the-Winner” theory suggests that lysogeny predominates at high microbial abundance and growth rates due to the benefits of preventing niche invasion from other viruses [36].

Very few studies have delved into the viral communities in and around brine pools and none have focused on viral life strategies. In addition to the pioneering study of viruses in brine pools by Corinaldesi et al. [37], metagenomic-based viral community characterization has only been carried out in two studies using shallow sequencing and only conducting preliminary analysis of viral diversity [38, 39], focusing mainly on prokaryotic viruses. Since viruses are potentially the main regulators of microbial populations around the brine pools, this knowledge gap is an important one to fill to gain insight into the limits of life and viral complexity. A key knowledge gap is the lack of a clear assessment of the diversity, host interactions, and life strategies of viruses in brine pools, which is needed for evaluating their roles in structuring the microbial communities and biogeochemical cycles. In this study, we sought to investigate active viral ecology, leveraging both abundance (metagenomic) and activity (metatranscriptomic) data to inform viral life strategies and patterns of infection across three distinct zones within the most recently discovered Red Sea brine pools, the NEOM pool system [7, 40]. By integrating these two types of data and using newly developed viral identification and host linkage tools, we seek to elucidate the complexities of viral-host interactions across a horizontal spatial gradient around and within the pools. In addition to this goal, we also categorize the eukaryotic virus community, showing active eukaryotic viral infections at a water depth of 1770 m in an extreme hypersaline environment. Leveraging all of this information, we also investigate patterns in viral activity across the study zones to shed light on how viral ecological dynamics might conform to or deviate from those observed in marine environments.

## Materials and methods

### Sample recovery

**Figure 1:**
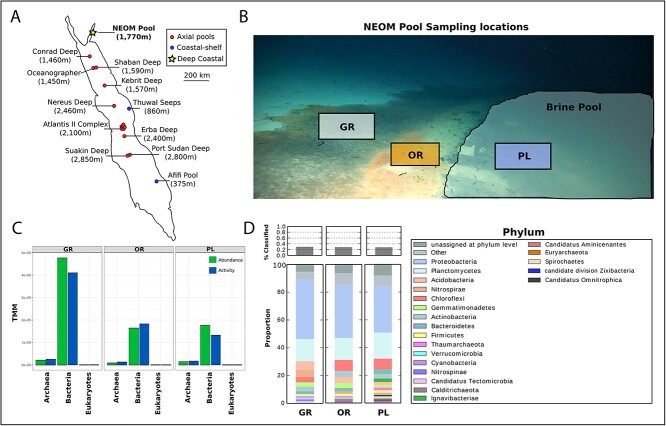
Sampling location and community composition; (**A**) a map of all discovered Red Sea brine pools, split into three categories based on location and depth, and (**B**) a picture taken from the ROV of the sampling locations showing the GR, OR, and PL; (**C**) abundance and activity of different kingdoms of life based on normalized metagenomic (abundance) and metatranscriptomic (activity) reads (TMM) mapped to contigs from each kingdom identified using Tiara; (**D**) relative abundance of different bacterial and archaeal phyla at each zone, and these proportions were determined using kaiju to assign taxonomy to trimmed metagenomic reads.

The most recently discovered brine pool cluster in the Red Sea, the NEOM brine pools, was discovered in 2020 [7] (Fig. 1A). These pools are distinct from previous Red Sea discoveries in that they were found only 2 km from the coast at a depth of 1770 m. This brine pool was found to have temperatures between 21 and 22 C, as well as a salinity of 160 PSU. [7].

The NEOM brine pool possesses three visually distinct microbial zones from which surficial sediment samples were collected using the manipulator arm from a remotely operated vehicle (ROV) These zones, representing microbial-dense areas around the pool, include a “gray zone” (GR) and “orange zone” (OR) that are shoreward of the pool, as well as a “pool zone” (PL) that was sampled from the sediment inside of the brine pool (Fig. 1B).

During a 2022 research mission conducted and facilitated by OceanX, samples were collected aboard the R/V OceanXplorer in the Gulf of Aqaba, Red Sea. Using an Argus Mariner XL ROV, a scoop of about 500 g of sediment were collected in each of the microbial zones surrounding the NEOM brine pool. Once secured aboard the research vessel, the sediment scoops were preserved in RNALater and kept at 4°C.

### Nucleic acid extraction and sequencing

The DNA extractions were performed using 2 grams of soil from each zone with the MagMax Microbiome kit from ThermoFisher following the manufacturer’s protocol. RNA was extracted with the RNA Powersoil Total RNA kit from Qiagen with a DNAse treatment. Samples were mixed prior to extraction.

For sequencing, metagenomic libraries were prepared with the xGen ssDNA & low-input DNA prep kit from IDT. For metatranscriptomic libraries, rRNAs were removed with a 5S/16S/23S Fastselect kit (Qiagen). These libraries were prepared with the Kapa Hyper Stranded mRNA library kit (Roche). All samples were pooled and run on a NovaSeq 6000 (Illumina) with V1.5 sequencing kits for 151 cycles. Generated FASTQ files were demultiplexed with bcl2fastq (v2.2) conversion software (Illumina). The generated paired-end reads were 150 bp in length.

Metagenomic and metatranscriptomic raw reads were trimmed using TrimGalore (v0.6.10) [41], and assembled using Megahit (v1.2.9) with the “-meta” flag [42]. Trimming and assembly statistics are present in a supplemental table (Table S1).

### Prokaryotic and eukaryotic abundance and diversity

In order to assess bacterial and archaeal abundances, bacterial and archeal contigs >10 kb were identified with Tiara (v1.0.3) [43] using a probability score cutoff of 0.65. Contigs were de-replicated at 95% average nucleotide identity (ANI) using dREP (v3.4.5) [44] resulting in a total of 266 243 bacterial contigs, 15 017 archaeal contigs, and 47 eukaryotic contigs. Trimmed reads were mapped to these contigs at 95% minimum identity using minimap2 (v2.17) [45] and coverage was determined by CoverM (v0.6.1) [46]. Raw counts were normalized using the trimmed mean of M-values (TMM) method in edgeR (v3.18) [47].

Bacterial diversity was determined using kaiju (v1.9.2) [48] with trimmed metagenomic reads from each zone. Taxonomy for each read was determined using the NCBI nr database [49] with a low abundance filter of 0.5 and a subsample percentage of 10. Eukaryotic contigs were classified to the phylum level using CAT (v5.3) [50].

### Prokaryotic virus taxonomy and diversity

Prokaryotic viruses were recovered from metagenomic datasets using the ViWrap (v1.2.1) pipeline with default parameters (−identify_method vb-vs) [51], which uses both VIBRANT (v1.2.1) [52] and VirSorter [53] to identify viral contigs, and vRhyme (v1.1) [54] to bin viral contigs into metagenome-assembled genomes (MAGs). Viral MAGs were classified as part of the pipeline using vContact (v0.11.3) [55] to find viruses of the nearest homology. After running the ViWrap pipeline, resulting viral MAGs were dereplicated at 95% average nucleotide identity (ANI) using dRep. This resulted in a total of 1184 viral genomes.

Trimmed metagenomic and metatranscriptomic reads were mapped to viral genomes and normalized the same way as for bacterial contigs. Prokaryotic viral diversity was calculated using the Shannon-Wiener index as part of the vegan R package (v2.6) [56]. The mean index was generated by 1000 bootstraps and a t-test was done on the differences of means to determine significance.

### Eukaryotic virus diversity and abundance

Viruses belonging to the phylum *Nucleocytoviricota* (NCLDV) were recovered from each zone’s metagenomic and metatranscriptomic dataset using the major capsid protein (MCP) marker gene. Briefly, proteins were predicted from each metagenome and metatranscriptome using prodigal-gv (v2.11) [57] and MCPs were recovered and identified using NCLDV-Markersearch [58]. MCPs recovered were then subjected to a 150aa length cutoff before being dereplicated at 95% amino acid identity using cd-hit [59]. These were aligned to reference sequences from the GVDB [58] using MAFFT (v7.520) [60] and trimmed using trimAl (v1.2) [61] with the “-gt 0.1” parameter. IQ-TREE (v1.6.12) [62] was used to build a maximum-likelihood tree of the alignment using the “LG + F + R10” model with 1000 bootstraps. The resulting tree was visualized using iTOL [63]. A total of 33 NCLDV MCPs were recovered using these methods.

Polintons, Polinton-like Viruses (PLVs), and Virophages are all united in their shared major capsid structure [64]. Using this information, a set of custom HMM profiles specific to diverse capsid proteins in these viruses were created as reported in Stephens et al. [65]. using HMMER3 (v3.4) [66] with an e-value cutoff of 1e-5. After applying a > 200 amino acid length cutoff, MCPs were dereplicated at 95% identity using cd-hit resulting in 183 total MCPs. These MCPs were aligned with reference sequences from Stephens et al. [65] and a phylogeny was created using the same method as for NCLDVs. RNA virus signatures were obtained using the RdRP-scan pipeline [67] to identify virus-specific RNA-dependent RNA polymerase (RdRp) homologs, and phylogenetic analysis was performed in a similar manner as above with references from the RdRP database [67]. A total of 243 RdRp were identified from all sites.

### Host prediction of prokaryotic viruses

As part of the ViWrap pipeline, iPHOP predicts hosts of prokaryotic viruses using a combination of host-based and virus-based methods [68]. A total of 36 virus-host linkages were predicted with a confidence score of over 90 using these methods. The resulting virus-host linkages were visualized using the Circos R package [69].

Additional hosts were predicted using CRISPR spacers inside of bacterial bins obtained from the datasets. Briefly, assembled contigs from each zone were binned using metabat2 [70], and resulting bins were classified with the GTDB-TK classify tool (v1.6.0) [71]. CRISPR spacers were identified in the bacterial bins using the web based CRISPR Recognition Tool (v1.1) [72] and then SpacePHARER (v5) [73] was used to match these CRISPR spacers to recovered virus MAGs. This method yielded a total of nine additional host predictions using an e-value cutoff of 1e-5.

### Active viral ecology and gene expression

After mapping reads to prokaryotic virus MAGs, these viruses were categorized based on their activity and abundance across zones. To elucidate trends in activity and abundance, viruses were assigned abundance and activity rankings for each zone. These rankings were used to first establish a “low-abundance, non-active” cutoff based on the point where the rank abundance curve flatlines for both abundance and activity. The remaining viruses were assigned an “Activity: Abundance” ratio that was used to cluster viruses by Euclidean distance. This hierarchical clustering yielded 3 main groups which were classified by investigating the average ratios of activity to abundance (RPKM).

Based on clustering, virus MAGs were separated into two groups to analyze differences in gene expression. A total of 178 “highly active” viruses were identified, and these were then compared to the 1006 viruses from other groups. Genes and proteins were predicted for both groups of viruses using prodigal [74]. Proteins were annotated using the Pfam [75], VOG [76], and eggNOG [77] databases with HMMER3 using an e-value cutoff of 1e−5. Metatranscriptomic reads were mapped to genes using CoverM at 95% minimum identity.

To analyze differences in gene expression between zones, viruses were grouped into zone-specific expression groups based on their TMM-normalized total expression at one zone compared to the others. A virus was deemed to be “zone-specific” if its total expression at one zone was greater than 10× higher than the other two zones combined, and it had a TMM value of greater than 1. The resulting groups were plotted in a 3D scatter plot using the scatterplot3d R package (v0.3–44) [78]. Once these zone-specific groups were established, the top 18 highly expressed genes in each of those groups were obtained and visualized using the pheatmap R package (v1.0.12) [79]. AMGs were obtained from VIBRANT [52] and were quantified in a similar manner to other genes.

### Jumbo phage identification and host analysis

Two jumbo phages were identified from the ViWrap outputs based on genome size (>200 kbp). Each of these jumbo phage genomes was annotated and plotted using pharokka [80]. Both jumbo phages were linked to host bacterial bins using CRISPR spacers and these bins were subject to metabolic investigation.

For manganese oxidation, the mnxG gene expression was used as a proxy for the entire process [81]. A custom hmm profile for mnxG was created using reference sequences of bacterial mnxG from the UniProt database [82]. This profile was used to search for mnxG genes in all bacterial bins using HMMER3 with an e-value cutoff of 1e−5. A similar method was used to recover the arsM genes for arsenic metabolism. After gene recovery, read mapping was performed in the same way as other gene mappings.

### Data availability

Raw reads are available on NCBI BioProject PRJNA1073546. Other data such as phylogenetic tree files, alignments, host predictions, protein annotations, phage genomes, code, and genome statistics are available on Figshare (https://figshare.com/projects/Active_prokaryotic_and_eukaryotic_viral_ecology_in_a_deep-sea_brine_pool/191886).

## Results

### Prokaryote abundance, activity, and diversity

We estimated the abundance of prokaryotic communities in the three study zones by mapping reads to the bacterial, archaeal, and eukaryotic contigs. This revealed an approximate 3-fold higher abundance of bacteria at GR (the site most shoreward of the pool) compared to the OR (intermediate zone) and PL (zone inundated with brine) (Fig. 1B, C). Bacteria were the most abundant taxa present at all zones, making up between 92% and 96% of the total reads mapped at each zone. Archaea made up around 4%–8% of total mapped reads at each zone and eukaryotes typically only made up ~0.03% (Fig. 1C). Of the 30% of bacterial reads that could be classified at each zone, the majority came from Proteobacteria and Planctomycetes across all zones.

### Abundance, activity, and diversity of prokaryotic viruses

We used the ViWrap pipeline to identify a total of 1184 prokaryotic viral genomes with various levels of completeness from the three samples. To determine zone-specificity and spatial niche partitioning of the prokaryotic viruses in the brine pool, we established a cutoff criterion (see Materials and methods). According to the established criteria, GR had the largest proportion of unique viruses with 319 and 316 zone-specific viruses in the metagenomic and metatranscriptomic data (Fig. S1A), respectively. Overall, abundance and activity followed each other closely (Fig. S2). Read mapping demonstrated a significantly higher diversity of viruses at OR (*P* < 2.2e−16) (Fig. S1B) and a larger number of viruses present at GR than the other zones following the trend in prokaryotic abundances (Fig. S1C).

**Figure 2:**
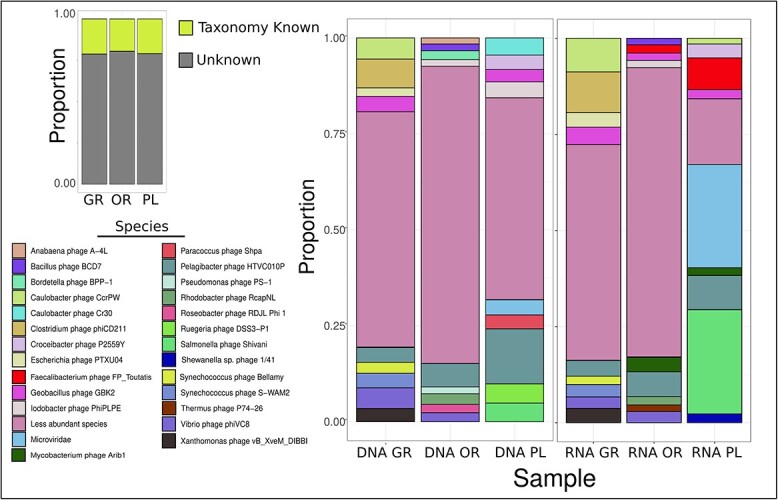
**Viral community composition across zones.** Viral homology at the species level was determined for 258 viruses, making up only ~25% of the total abundance and activity of all viruses in the community. The proportion of the top viral species for each zone is shown for metagenomic (DNA) and metatranscriptomic (RNA) data, with less abundant species being grouped together. Proportional abundance within a zone is determined from RPKM normalization, taking genome length and reads mapped as normalization factors.

Out of the 1184 viral genomes, we were able to assign taxonomic labels to 258 (22%). These viruses made up around 25% of total viral abundance and activity at each zone, demonstrating the presence of a large viral dark matter (Fig. 2). Of the viruses with taxonomic assignment, differences between zones emerged as OR had the highest number of less abundant species, reflected in the higher diversity index.

### Eukaryotic virus abundance, activity, and diversity

**Figure 3:**
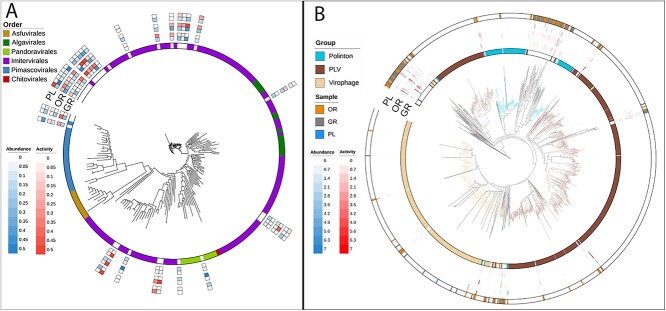
**Phylogenetic diversity of eukaryotic viruses.** (**A**) a phylogeny of the giant virus (NCLDV) major capsid protein (MCP) with reference sequences from the GVDB. Reads were mapped to MCPs and abundance and activity are represented for each zone as log normalized TMM values. (**B**) a phylogenetic tree showing identified MCPs from Polintons, PLVs, and Virophages from all zones as well as their abundance. Reference sequences were obtained from Stephens et al. [65]. Reads were similarly mapped to MCPs and abundance and activity of individuals is shown in the heatmap. The outer ring represents the site where the MCP was recovered from.

Viruses from the phylum NCLDV, are abundant in the Earth’s oceans and have been found to inhabit a range of different environments as well as modulate host metabolism and genome evolution [83, 84]. The main hosts for these viruses include microeukaryotes such as unicellular algae and protists. Searching for MCP deriving from *NCLDVs* yielded a total of 33 MCPs in our brine pool data. Most of these MCPs belong to viruses in the *Imitervirales* order, but members of *Pandoravirales* and *Pimascovirales* were also identified (Fig. 3A). NCLDVs were present across zones, with OR having the largest total abundance (Fig. S3A).

Virophages, Polintons, and PLVs are viruses or virus-like transposable elements that have a shared evolutionary origin and can integrate into eukaryotic genomes or co-infect with NCLDVs. We used a set of MCP hidden Markov profiles. This search revealed 183 viruses from these groups and phylogenetic analysis revealed that most brine pool sequences formed a new clade within the Polintons, with some also forming a deeply rooted group within the PLVs (Fig. 3B). Members of these putative new clades were highly abundant and active across all zones.

RNA viruses were also found to be active and abundant across sites, largely forming a unique clade within phylum Pisuviricota (Fig. S4).

### Prokaryotic and putative eukaryotic viral hosts

**Figure 4:**
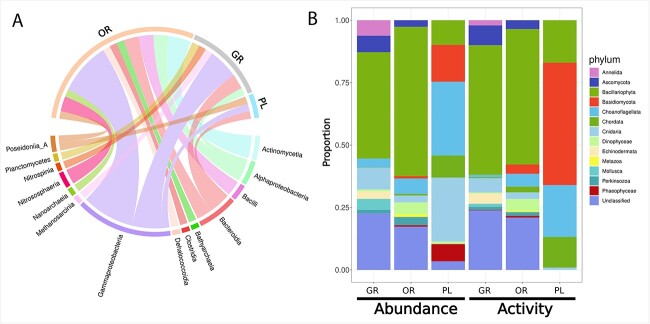
**Predicted and potential hosts for prokaryotic and eukaryotic viruses.** (**A**) Virus-host predictions for prokaryotic viruses were done using iPHOP for all zones. A total of 36 host predictions were made across zones and band thickness represents more hosts from a given bacterial class or zone. (**B**) the potential eukaryotic host pool was created through mapping reads to confirmed eukaryotic contigs obtained from Tiara. These contigs were classified to the phylum level using CAT and proportions represent RPKM values for each zone.

Despite the large diversity of novel viruses in these zones, we were only able to predict hosts for 36 of these viruses potentially infecting prokaryotes. Most of these predicted hosts were members of the class Gammaproteobacteria, consistent with Proteobacteria being the most abundant phylum across zones (Fig. 4A).

We also identified the eukaryotic community in the brine PLs that potentially serve as the host pool of the NCLDVs present. Using Tiara, we identified 357 eukaryotic contigs and reads were mapped to these contigs to obtain abundance and activity levels. Among the eukaryotic taxa present, the most abundant and active phylum around the edges of the pool was Bacillariophyta, making up over 50% of the abundance and activity at the OR and having similar high abundance at the GR (Fig. 4B). Other eukaryotes such as Choanoflaggelata, Cnidaria, Ascomycota, and Mollusca were also found.

### Active infection ecology of brine pool viruses

**Figure 5:**
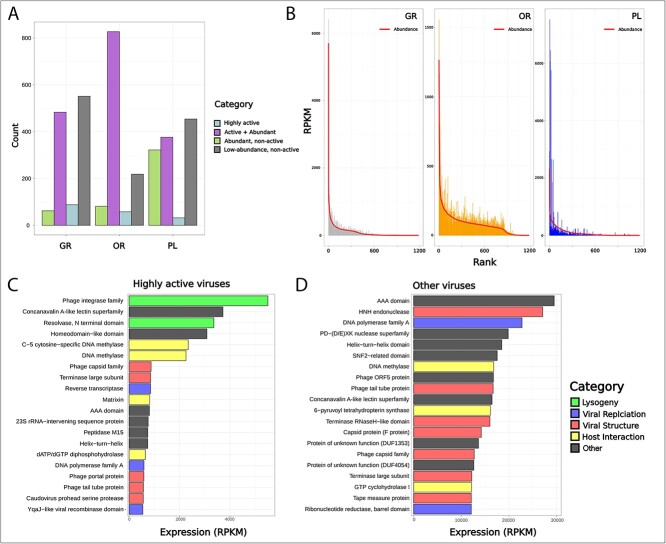
**Active viral ecology and gene expression.** (**A**) Viruses from each zone were categorized as either being “highly active”, “active + abundant”, “abundant, non-active”, and “non-abundant, non-active” based on hierarchical clustering of activity and abundance ratios. (**B**) Rank abundance curves for each zone with the line representing metagenomic (abundance) read mapping and the bars representing metatranscriptomic (activity) data. Both read counts were normalized with RPKM. The gene expression profiles of (**C**) highly active and (**D**) other viruses. The top 20 genes (derived from sum RPKM) from each group are displayed in descending order. Categories were created based on gene annotations found within the PFAM database.

To further delve into the dynamics and functional potential of viruses in the brine pool, we separated the viral populations into four categories based on hierarchical clustering of individual virion rank activity to rank abundance ratios (Fig. S5) (see Methods). GR had the highest number of “low-abundance, non-active” viruses (*n* = 549) but also the highest number of “highly active” viruses (*n* = 88) (Fig. 5A), while OR had the highest number of “active + abundant” viruses (*n* = 827), making up 90% of viruses in this zone.

Analysis of the viral community’s rank abundance curves for both abundance and activity provides further insight into the differences in viral ecology across zones, confirming what was seen in (Fig. 5A, B). For all zones, there was a sharp drop-off in both activity and abundance with the rank abundance curve showing a strong left-skew, demonstrating that a few viruses at each zone made up a large portion of the activity and abundance for the given zone. However, this drop-off was less severe for OR, and the rank abundance curve showed a more even distribution.

A total of 178 “highly active” viruses were identified across zones. In order to elucidate differences in gene expression patterns between these highly active viruses and other groups, the activity of the genes in these viruses was assessed. Interestingly, while many genes crucial to viral replication and interaction with their host were similarly expressed across groups, the highly active viruses showed genes involved in lysogeny to be the most highly expressed (Fig. 5C). This was not true of other viral categories, as genes related to viral replication and structure were among the most highly expressed (Fig. 5D).

### Stratification of gene expression and AMGs across zones

**Figure 6:**
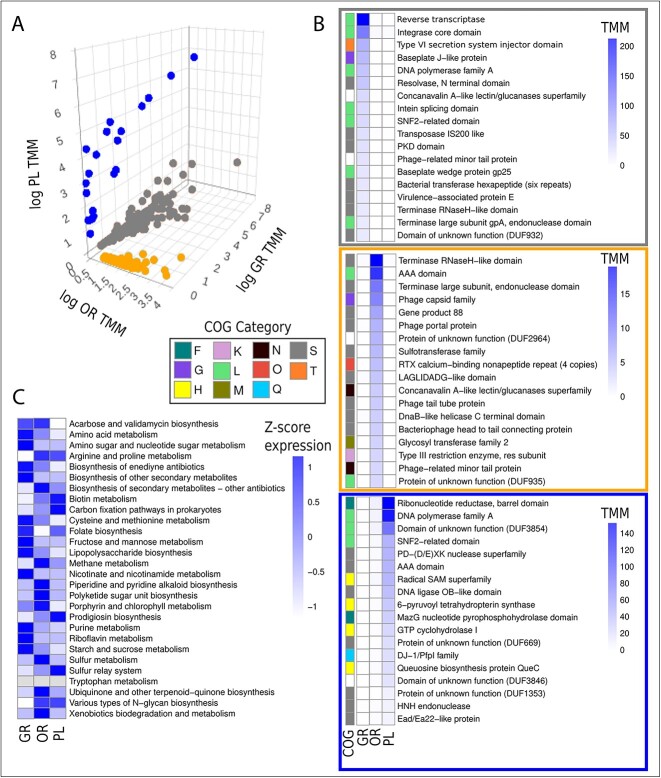
**Zone specific marker genes and AMGs.** (**A**) Viruses that were deemed “exclusive” to a certain zone (see Fig. 2) were displayed on a three-dimensional scatter plot with axes representing the log TMM of activity data from each zone. (**B**) within each zone-specific cluster, the top 18 genes (by sum TMM) within that zone were plotted. COG categories were assigned based on eggNOG annotations [F: Nucleotide metabolism, G: Carbohydrate metabolism, H: Coenzyme metabolism, K: Transcription, L: Replication and repair, M: Cell wall/membrane biogenesis, N: Cell motility, O: Post-translational modification, Q: Secondary metabolite biosynthesis, S: Unknown, T: Signal transduction]. (**C**) AMGs were obtained from viruses at each zone and reads were mapped to calculate activity. This heatmap shows across-zone normalized Z-scores from TMM values.

To further understand differences in expression patterns between viral communities at the different zones, viruses specific to each zone were obtained (Fig. 6A) (see Materials and methods). Once viruses were grouped into the three zone-specific groups, most expressed genes of that group were investigated to gain insight into the potential activity and life strategy of viruses at each zone (Fig. 6B). GR had reverse transcriptase, and integrase as its most expressed genes. Other notable genes of interest in this list are resolvase, a gene known to be involved in the lysogenic cycle, as well as a virulence-associated protein. An independent read mapping of phage integrase genes across zones (normalized for total viral activity and library sizes) confirmed this finding as GR had a level of integrase expression nearly 3.5x higher than OR, and over 13x higher than PL (Fig. S6).

OR has terminase-related genes and an AAA domain-encoding gene as most highly expressed along with the phage capsid. PL had genes involved in replication among the most highly expressed ones such as ribonucleotide reductase, and DNA polymerase A.

In total, 101 different AMGs were recovered from the viral genomes, belonging to 28 different metabolic pathways (Fig. 6C). Some of these AMGs recovered were homologous to genes from pathways involved in the biosynthesis of important biomedical compounds such as acarbose, validamycin, and enediyne antibiotics. Other interesting pathways include the recovery of genes involved in methane (mgsA) and sulfur (cysH) metabolism as well as xenobiotics degradation (guaA).

### Jumbo phages infecting metabolically relevant hosts

**Figure 7:**
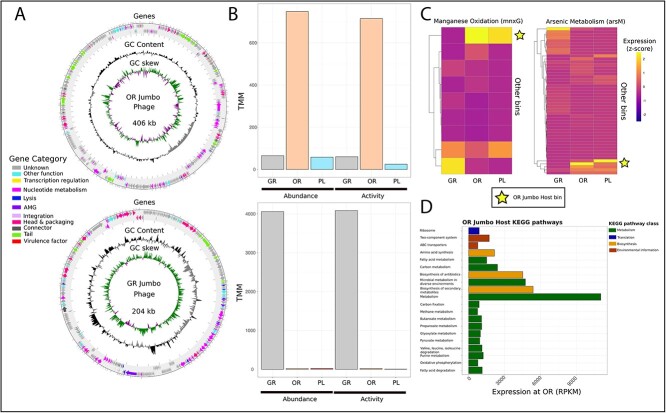
**Jumbo phages and their hosts**. (**A**) Genome maps of two identified jumbo phages were created using pharokka. GC skew, GC content, and gene categories were displayed on each track. (**B**) Reads were mapped to each jumbo phage genome and normalized using TMM across zones. Abundance and activity for each phage at each zone is represented here. The metabolic profile of the CRISPR-linked host of the OR jumbo phage is displayed through both a targeted and general approach. (**C**) Manganese oxidation and arsenic metabolism gene expression from all bacterial bins was calculated through read mapping to recovered genes and TMM normalization. Normalized reads were displayed as z-scores, normalized for within zone comparison to parse out key players in each process. The stars represent the OR jumbo host bacterial bin’s expression of these respective genes. (**D**) Reads were mapped to genes predicted from the OR host bacterial bin and normalized using RPKM. KEGG pathways were put into larger self-determined categories based on similar functions.

Our genome binning approach recovered the genomes of two jumbo phages (phages with genome sizes larger than 200 kbp (Fig. 7A). These two jumbo phages showed high abundance and activity, ranking within the top 1% in terms of activity and abundance at their respective zones with high zone specificity (Fig. 7B, S7A) as well as unique gene expression patterns (Fig. S7B).

Through CRISPR spacer matching between these jumbo phages and bacterial bins recovered from the brine pool, we were able to match both jumbo phages with a potential host. While the potential host for the GR jumbo phage was a Proteobacteria with a partial genome, the host for the OR jumbo phage was found to be a metabolically active bacterium from the phylum *Myxococcota*. This bacterium was found to be the highest contributor among bacterial bins to manganese oxidation and arsenic metabolism at this zone, having the highest expression of genes involved in these pathways (Fig. 7C). It also contained genes involved in methanogenesis and biosynthesis of antibiotics that are expressed (Fig. 7D).

## Discussion

### Spatial stratification of the bacteria and virus communities

Through leveraging a multi-omics approach, we were able to gain insight not only into the diversity and abundance of viruses but also into their active infection dynamics, gene expression, and putative life strategies. It was surprising that even across spatial distances of just a few meters, bacterial and viral communities were stratified, displaying large differences in both diversity and abundance. Previous studies have demonstrated vertical stratification of brine pool bacterial [23] communities, but here we show that within short distances in the same pool, the viral community varies dramatically. Our data are evidence that stratification is not only caused by the oxic/anoxic gradient but also by an underlying nutrient or physiochemical gradient along the edge of the pool. Other studies have characterized potential gradients of methane, salinity, and temperature around brine pools which could be contributing to the stratification seen here [85–87].

Bacterial abundance and viral abundance followed similar patterns, with GR having the highest abundance of both. A potential explanation for these patterns comes from the fact that while the brine is a nutrient-rich particle trap, the brine is also a hypersaline pool that restricts the growth of many microbial species. The brine has been reported to splash outside the pool with minor disturbances and is episodically disturbed by underwater landslides [7, 40]. GR could be situated in the perfect area that can receive vital nutrients from the brine pools without too much direct contact with the harmful brine, harboring 3x more bacteria than other zones closer to the pool. In this way, the brine pool ecosystem can be thought of like an intertidal ecosystem.

### The viral dark matter of the NEOM brine pools

Previous studies of viruses in the brine pools have found high levels of unclassified prokaryotic viruses within the pools [38, 39] and our study confirmed this, with a large proportion of viruses showing no homology to known representatives. In addition to finding a large diversity of prokaryotic viruses, novel eukaryotic viruses were identified around the pool. Previously, viruses of the phylum NCLDV have been found in metagenomic data from the brine pools [38], but the conclusion was that these viruses were most likely “pickled” (preserved in the hypersaline brine but not active) and not actively infecting hosts. Here, through leveraging metatranscriptomic data, we demonstrate that NCLDVs are not only abundant in the brine pools, but are also active, possibly infecting eukaryotic hosts such as Bacillariophyta, Mollusks, Dinoflagellates, Phaeophytes, or fungi seen in the eukaryotic host pool [88–95].

In addition to NCLDVs, we identified many Polintons, PLVs, and virophages in and around the brine pool. These evolutionarily related groups [96] all infect eukaryotic hosts and are widely abundant in aquatic ecosystems and within the genomes of eukaryotes [96, 97]. The presence and activity of virophages also confirm the activity of NCLDVs in the brine pools as virophages typically depend on NCLDVs for co-infection [98]. The formation of a novel clade within the Polintons shows the potential untapped viral diversity within extreme environments.

### Active viral ecology at the brine pool

Besides characterizing the virosphere of the brine pools, we also sought to gain insight into virus-host dynamics and life strategies in an extreme environment, something that is left out of previous brine pool viral studies that only provide a diversity analysis [38]. Through leveraging metagenomic and metatranscriptomic data, we were able to show that a small number of viruses at both GR and PL were contributing to most of the total viral activity and abundance at these zones. This is reflected in viral diversity as it was significantly lower in these two zones compared to OR, which displayed many viruses that were both active and abundant. These differences can be explained in part by the adherence to the viral “bank” model which suggests that at a given time only a fraction of the viruses in a zone are active, while the majority are inactive [99]. While GR and PL seem to obey the model, the OR deviates, demonstrating the possibility for the breakdown of generalized viral strategic models when applied to specific individual environments.

Our categorization of virus communities based on their level of activity helped to elucidate the infection strategies of the viruses that were highly active relative to their abundance. Analysis of gene expressions revealed that many viruses in this highly active category were most likely temperate phages. Further exploration into lysogenic markers such as integrase showed that most of these temperate phages are likely most active at GR, furthest away from the brine. This zone has the highest bacterial population, which seems to be consistent with the “Piggyback-the-Winner” hypothesis that predicts temperate strategies become more prevalent with higher bacterial abundance [36].

In addition to elucidating viral infection strategy, the metatranscriptomics data also gave insight into the potential metabolic impacts these viruses may have on their hosts. Previous studies have found high methane concentrations in several brine pools [100–102]. Here we find that viruses around the brine pool infect key methanogenic bacteria and archaea such as Methanosarcina [103] as well as contain AMGs within their genomes that aid in methane metabolism (msgA). These findings suggest that viruses play a key role in regulating and modulating msgA in the brine pool environment as has been suggested in other environments [104, 105].

Brine pools are unique environments that have been shown to be enriched in manganese and arsenic [7, 106]. This enrichment compared to the surrounding sediment allows for specific prokaryotic adaptations to utilize these trace elements [7, 106, 107]. Through direct CRISPR spacer linkage, we identified a jumbo phage infecting one of these key microbial players in manganese oxidation and arsenic metabolism. This linkage presents the possibility that deep sea jumbo phages could be regulating key biogeochemical cycles important to life in the brine pools.

### A conceptual model of brine pool virus ecology

**Figure 8:**
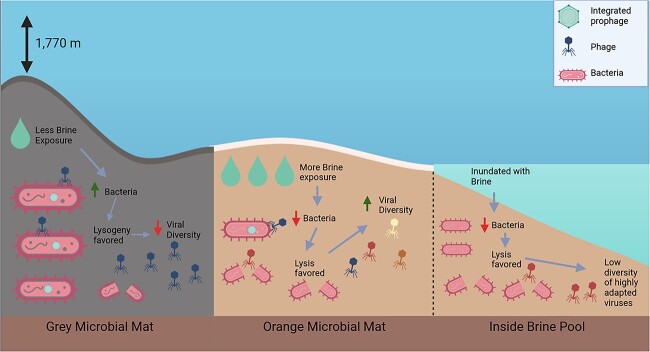
**A conceptual model of brine pool virus ecology.** An artistic rendition of our hypothesized prokaryotic viral ecology in the brine pool. Created with BioRender.com.

Based on the patterns in viral diversity and ecological strategies observed in our study, we propose a hypothetical framework to explain the complexities of viral ecology within brine pools (Fig. 8). This framework is grounded on the premise that brine pools behave similarly to intertidal habitats in that they show spatial stratification due to sediment physicochemical differences and the splashing of nutritious but potentially hazardous brine out of the pool [40]. The combination of these two factors creates unique microhabitats, allowing for unique viral-host interactions and abundance patterns across a small scale of stratification.

The GR, which is furthest away from the center of the pool but still close enough to benefit from nutrients delivered by it, shows the highest bacterial abundance. This high bacterial abundance leads to an increase in lysogeny among the viral population, as these are key factors in predicting lysogeny in the “Piggyback the winner” model [36]. The higher presence of lysogeny at this zone causes an increase in “superinfection exclusion”, a phenomenon in which lysogeny of one virus prevents others from infecting the same host [108, 109]. This exclusion may explain why in this zone, there are a lower diversity of viruses and only a small proportion of the viral community that contributes to overall viral activity and abundance.

At the OR, right at the interface of the brine pool, microbes are likely exposed to the brine daily either through soil penetration or splashing [7, 40]. This increased exposure may be responsible for observed lower bacterial populations, favoring viruses undergoing the lytic cycle [110].

Inside the brine pool, a zone characterized by full hypersaline and anoxic conditions, lower bacterial abundance is observed. Similar to the OR, lytic viruses are favored but unlike the OR, there is a lower viral diversity due to the potential selection of fewer highly adapted viruses such as Microviridae members.

## Conclusion

Overall, our study aimed to address the knowledge gap regarding the viral community in extreme brine pool environments. Through integrating metagenomic and metatranscriptomic data, we were able to elucidate viral strategies and infection dynamics at a spatial scale in a brine pool. Our data provide valuable insights into the viral community within these pools and set the stage for further exploration and characterization of the physicochemical parameters that potentially drive the observed stratification in viral diversity and ecological strategies. Results from this research will be instrumental in generating further hypotheses and exploration into these unique environments with flourishing life around them.

## Supplementary Material

Supplementary_file_updated_ycae084
